# Macrophage C1q contributes to pulmonary fibrosis by disturbing the metabolism of alveolar epithelial cells

**DOI:** 10.1002/ctm2.70341

**Published:** 2025-05-22

**Authors:** Fenja Prüfer, Beatrix Steer, Eva Kaufmann, Peter Wolf, Barbara Adler, Martina Korfei, Andreas Günther, Melanie Königshoff, Heiko Adler

**Affiliations:** ^1^ Comprehensive Pneumology Center, Research Unit Lung Repair and Regeneration, Helmholtz Zentrum München ‐ German Research Center for Environmental Health Munich Germany; ^2^ University Hospital Grosshadern Ludwig‐Maximilians‐University Munich Munich Germany; ^3^ German Center for Lung Research (DZL/CPC‐M) Munich Germany; ^4^ Institute of Asthma and Allergy Prevention, Helmholtz Zentrum München, German Research Center for Environmental Health Neuherberg Germany; ^5^ INCYTON GmbH Planegg Germany; ^6^ Max von Pettenkofer Institute & Gene Center, Virology, Faculty of Medicine Ludwig‐ Maximilians‐University Munich Munich Germany; ^7^ Department of Internal Medicine Justus‐Liebig‐University Giessen Giessen Germany; ^8^ German Center for Lung Research (DZL), Universities of Giessen and Marburg Lung Center (UGMLC) Giessen Germany; ^9^ Department of Medicine University of Pittsburgh Pittsburgh Pennsylvania USA; ^10^ Walther Straub Institute of Pharmacology and Toxicology Ludwig‐Maximilians‐University Munich Munich Germany

1

Dear Editor,

Idiopathic pulmonary fibrosis (IPF) is a devastating interstitial lung disease, driven primarily by damage and dysfunction of type‐II alveolar epithelial cells (AECII). Since there is still no cure for IPF, new therapeutic approaches are desirable. In this study, we identified the complement component C1q as a source of disturbance of AECII metabolism.

When analyzing publicly available data,^1^ we found that C1q mRNA expression is upregulated in IPF patients (Figure S1). Therefore, we analyzed bronchoalveolar lavage fluids (BALF) of IPF patients for the presence of C1qA. Compared to controls, significantly increased levels of C1qA were present in BALF of IPF patients (Figure 1A). To investigate the role of C1q in fibrosis development, we used a virus‐induced mouse model of IPF.^2^ First, we determined C1qA gene expression in lungs of both control (wild‐type) and fibrosis‐prone (interferon [IFN]‐γR‐/‐) mice, analyzing uninfected mice and mice 14 days (acute inflammation phase), around 45 days (early fibrosis phase), and around 100 days (fibrosis phase) after infection with murine gammaherpesvirus 68 (MHV‐68). C1qA was highly expressed in the acute inflammation phase in both mouse strains. It subsequently declined over time in wild‐type mice whereas the decline was much less pronounced in IFN‐γR‐/‐ mice (Figure 1B). No differences were observed when comparing whole lung tissue protein levels (Figure 1C). However, mice with lung fibrosis (IFN‐γR‐/‐) showed significantly elevated C1qA protein in BALF (Figure 1D). This was due to local production and secretion as in mice with increased C1qA in BALF (Figure S2A), C1qA in serum remained constant (Figure S2B). Higher C1qA gene expression in fibrotic mice was also observed in other fibrosis mouse models when analyzing publicly available data (Figure S3).

**FIGURE 1:**
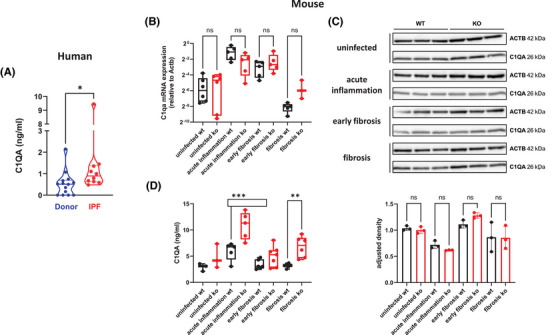
Increased levels of C1QA in BAL fluids of both IPF patients and fibrotic mice. (A) C1QA in BAL fluids of IPF patients was determined by ELISA. The data are presented as violin plots, with median and quartiles indicated. Each symbol represents one individual (Donor: *n =* 14; IPF: *n =* 10). The asterisk indicates a statistically significant difference (Mann‐Whitney test). (B) Lungs of uninfected (*n =* 6 per group) and MHV‐68 infected C57BL/6 (WT) and IFN‐γ R‐/‐ (KO) mice were harvested during acute inflammation (*n =* 5 per group), early fibrosis (*n =* 5 per group) and fibrosis (*n =* 3‐5 per group). RNA was isolated from the tissue and C1qA expression was analyzed by qRT‐PCR, normalized to β‐actin (Actb), and displayed relative to Actb. Results are shown as Box‐Whisker‐Plots, and each symbol represents an individual mouse. ns: not significant (One‐way ANOVA). (C) Lungs of uninfected and MHV‐68 infected C57BL/6 (WT; *n =* 3) and IFN‐γ R‐/‐ (KO; *n =* 3) mice were harvested during acute inflammation, early fibrosis and fibrosis. C1QA and β‐actin (ACTB) protein levels were analyzed by Western blot (upper panel). Band density was measured with ImageJ, adjusted to β‐actin levels, and displayed as adjusted density (lower panel). Each symbol represents an individual mouse, and the columns represent the mean ± SD. ns: not significant (Kruskal‐Wallis test). (D) BAL fluids of uninfected (*n =* 3 per group) and MHV‐68 infected C57BL/6 (WT) and IFN‐γ R‐/‐ (KO) mice were collected during acute inflammation (*n =* 5 per group), early fibrosis (*n =* 5‐6 per group) and fibrosis (*n =* 6 per group). C1QA concentration was measured by ELISA. Results are shown as Box‐Whisker‐Plots, and each symbol represents an individual mouse. The asterisks indicate a statistically significant difference (One‐way ANOVA).

To determine the cellular source of C1q in the lung, we analyzed C1q gene expression in publicly available single‐cell RNA sequencing data (Figure S4). In human lungs, macrophages are the main producers of C1qA, C1qB and C1qC. We immuno‐stained sequential slices of murine lungs with the macrophage marker F4/80 and anti‐C1qA (Figure S5A), and sequential slices of human lungs with anti‐C1qA, the macrophage marker CD68 and the AECII marker proSPC (Figure S5B). In both cases, macrophages stained positive for C1qA. To further analyze C1q production by macrophages, we polarized MH‐S cells (a murine alveolar macrophage cell line) into either M1 or M2 macrophages. Treatment with LPS + IFN‐γ led to an increase in Nos2 expression, an M1 marker, while treatment with IL‐4 resulted in increased Arg1 expression, an M2 marker (Figure S6A). Polarization into M2 macrophages did not increase cell‐associated C1qA (Figure S6B) but significantly increased C1qA in the supernatant (Figure S6C).

We hypothesized that secreted C1qA might affect other cells in a paracrine fashion and focused on AECII. MLE‐12 cells, a well‐established murine AECII line, were treated with native human C1q that also reacts with murine cells.^3^ We observed a dose‐dependent reduction in MTT (3‐(4,5‐dimethylthazolk‐2‐yl)‐2,5‐diphenyltetrazolium bromide)‐assay activity (Figure 2A), suggesting that C1q induces cell death of AECII since the MTT‐assay is commonly used as a measure of cell viability.^4^ Since we did not observe a corresponding increase of lactate dehydrogenase (LDH)‐activity (an indicator of necrosis) in the supernatants of the treated cells (Figure 2B), we speculated that C1q induces non‐necrotic cell death of AECII. As apoptosis of AECII is a main characteristic of IPF, we conducted an Apoptosis and Necrosis Assay. C1q did neither induce apoptosis (Figure S7A) nor necrosis (Figure S7B). We further confirmed the absence of apoptosis by both the Caspase‐Glo 3/7 Assay (Figure S7C) and Western blot for cleaved PARP (Figure S7D). Next, we analyzed other known cell death pathways including ferroptosis, autophagy‐dependent cell death, necroptosis, pyroptosis and parthanatos. We treated MLE‐12 cells with C1q in the presence or absence of specific cell death inhibitors, and subsequently determined cell viability by MTT‐assay, a strategy successfully applied by others.^5^, ^6^ None of the inhibitors was able to reverse the C1q‐induced reduction in MTT‐assay activity (Figure S8). To support these findings, we determined the gene expression of characteristic cell death‐associated genes including Bax, Bcl‐xl, Acsl4, RIPK3 and NLRP3 (Figure S9). We did not observe significant differences between control‐ or C1q‐treated MLE‐12 cells.

**FIGURE 2:**
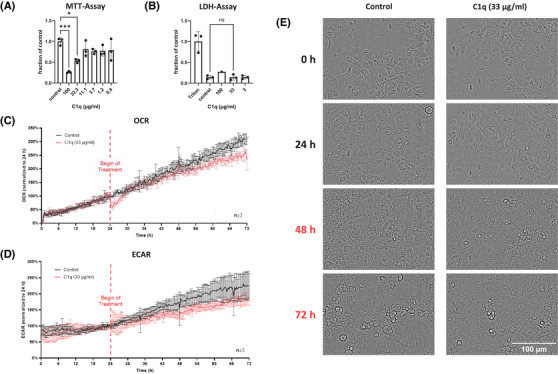
C1QA induces metabolic decline of AECII. (A) MLE‐12 cells were treated with C1q in concentrations ranging from 100 µg/mL to 0.4 µg/mL in a 3‐fold dilution series. Culture medium served as control. Metabolic activity was measured 48 h after treatment with an MTT assay. Values were normalized to the mean of the control group and are presented as a fraction of the control. Each symbol represents an individual experiment, and the columns represent the mean ± SD. The asterisks indicate a statistically significant difference (One‐way ANOVA). (B) MLE‐12 cells were treated with different concentrations of C1q. Culture medium and Triton X 100 served as negative and positive controls. Cytotoxicity was measured 48 h after treatment by measuring LDH concentration with an LDH assay. Values were normalized to the mean of the control group and are presented as a fraction of control. Each symbol represents an individual experiment, and the columns represent the mean ± SD. ns: not significant. (C–E) MLE‐12 cells were pre‐measured (baseline) for 24 h and then treated with C1q (33 µg/mL) or with diluent (medium) as a control as indicated. Metabolic rates (OCR [panel C] and ECAR [panel D]) were determined during the next 48 h. The data shown are the means and standard deviations of 3–4 replicates. Microscopic images of all wells were taken periodically throughout the entire experiment, and exemplary images from the indicated time points are shown in panel E.

Since our data suggested that the reduction in formazan formation (MTT‐assay) upon C1q treatment is not due to AECII death, we performed multiparametric monitoring of cellular metabolic responses, continuously measuring oxygen consumption rate (OCR) and extracellular acidification rate (ECAR) and simultaneously performing periodic microscopic imaging using the CYRIS FLOX analysis platform (INCYTON GmbH, Planegg, Germany). Treatment of MLE‐12 cells with a moderate dose of C1q (33 µg/mL) resulted in a significant reduction of both OCR (Figure 2C) and ECAR (Figure 2D) (C1q vs. control: *p* < 0.05; Mann‐Whitney test). In contrast, the morphological integrity of the cells was not affected (Figure 2E).

Finally, we applied a C1q inhibitory peptide^7^ that has already been successfully used in vivo in a mouse model of chronic hepatitis^8^ to investigate fibrosis development in the presence or absence of the peptide. IFN‐γR‐/‐ mice were left uninfected or were infected with MHV‐68. From day 44 (= early fibrosis phase) after infection, the infected mice were either treated i.p. with 2J peptide (2 mg/kg) or with vehicle control twice a week as described by others^8^ until day 85 (= fibrotic phase) after infection. Treatment with the C1q inhibitory peptide significantly improved peripheral oxygen saturation (SpO_2_) (Figure 3A), and considerably prevented fibrosis development as determined by Hematoxylin/Eosin‐ and Masson‐Goldner trichrome‐staining of lung sections (Figure 3B,C; Figure S10) and by measurement of mean septal thickness (an indicator of fibrosis) (Figure 3D).

**FIGURE 3:**
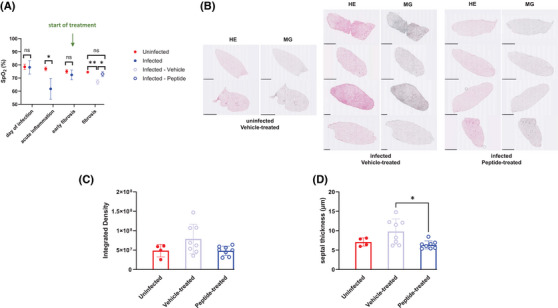
Inhibition of C1q in vivo reduces fibrosis development. IFN‐γ R−/− (KO) mice were left uninfected (*n =* 2) or were infected with MHV‐68 (*n =* 8). Beginning at day 44 (= early fibrosis phase) after infection, the infected mice were either treated i.p. with 2J peptide (2 mg/kg) (*n =* 4) or with vehicle control (*n =* 4) twice a week. (A) SpO_2_ was determined at the indicated time points using a Pulse Oximeter for mice (MSTAT‐JR, Kent Scientific Corporation). Data shown are means ± SD. The asterisks indicate a statistically significant difference (unpaired, two‐sided t‐test). ns: not significant. (B) At day 85 after infection (= fibrosis phase), all mice were sacrificed, and the lungs were prepared for histology. Representative Hematoxylin/Eosin (HE)‐ and Masson‐Goldner (MG) trichrome‐stained sections are shown. Scale bars indicate either 1 mm or 2.5 mm. (C) Densitometric quantification of sections from left and right lungs using ImageJ. (D) Measurement of the length of mean septal thickness (µm) in HE‐stained lung sections from left and right lungs. This measurement was performed by a researcher blinded to the study groups. In panels (C) and (D), each symbol represents the value of an individual lung section, and the columns represent the mean ± SD. The asterisk indicates a statistically significant difference (Kruskal‐Wallis test).

In summary, we identified C1q, produced by macrophages, as an inducer of a metabolic disorder of AECII, emphasizing the importance of macrophage—epithelial cross‐talk for the development of pulmonary fibrosis, and propose C1q as a potential new therapeutic target.

## AUTHOR CONTRIBUTIONS

Fenja Prüfer performed experiments and analyzed and interpreted the data. Beatrix Steer performed experiments. Eva Kaufmann performed experiments and analyzed and interpreted the data. Peter Wolf performed experiments and analyzed and interpreted the data. Barbara Adler discussed and interpreted the data, and reviewed and revised the final manuscript. Martina Korfei performed experiments and analyzed and interpreted the data. Andreas Günther discussed and interpreted the data. Melanie Königshoff discussed and interpreted the data. Heiko Adler supervised the project, designed the experiments, analyzed the data and wrote and edited the paper.

## CONFLICT OF INTEREST STATEMENT

Peter Wolf is an employee of the company INCYTON GmbH (Germany), which developed and distributes the utilized analysis platform CYRIS FLOX. All other authors declare no conflict of interest.

## FUNDING INFORMATION

This work was funded by the FöFoLe‐program of the LMU Munich as well as by the German Center for Lung Research (DZL).

## ETHICS STATEMENT

The study was approved by the local ethics committee of the Ludwig‐Maximilians‐University Munich, Germany (Projects 333‐10, 455‐12).

## PATIENT CONSENT STATEMENT

Written informed consent was obtained for all study participants. The animal study protocol was approved by the government of Upper Bavaria and performed in compliance with the German Animal Welfare Act.

## Supporting information



Supporting Information

## Data Availability

The data presented in this study are available upon request from the corresponding author.
