# Supplementary material for: Macrophage C1q contributes to pulmonary fibrosis by disturbing the metabolism of alveolar epithelial cells
Source: Clin Transl Med. 2025 May 22;15(5):e70341. doi: 10.1002/ctm2.70341 (PMC12098955; doi:10.1002/ctm2.70341)
Supplement: Supplementary file 1 — Supporting Information [file CTM2-15-e70341-s001.pdf]

Supplementary Information

Macrophage C1q contributes to pulmonary fibrosis by disturbing the metabolism of alveolar epithelial cells

Fenja Prüfer [1], Beatrix Steer [2], Eva Kaufmann [1], Peter Wolf [3], Barbara Adler [4], Martina Korfei [5], Andreas Günther [5], Melanie Königshoff [6], and Heiko Adler [2, 7 *]

[1] Comprehensive Pneumology Center, Research Unit Lung Repair and Regeneration, Helmholtz Zentrum München - German Research Center for Environmental Health, Munich, Germany, and University Hospital Grosshadern, Ludwig-Maximilians-University Munich, Munich, Germany; Member of the German Center for Lung Research (DZL/CPC-M), Munich, Germany

[2] Institute of Asthma and Allergy Prevention, Helmholtz Zentrum München, German Research Center for Environmental Health, Neuherberg, Germany; Member of the German Center for Lung Research (DZL/CPC-M), Munich, Germany

[3] INCYTON GmbH, Am Klopferspitz 19A, 82152 Planegg/Martinsried, Germany

[4] Max von Pettenkofer Institute & Gene Center, Virology, Faculty of Medicine, Ludwig-Maximilians-University Munich, Munich, Germany

[5] Department of Internal Medicine, Justus-Liebig-University Giessen, Giessen, Germany; German Center for Lung Research (DZL), Universities of Giessen and Marburg Lung Center (UGMLC), Giessen, Germany

[6] University of Pittsburgh, 6614, Department of Medicine, Pittsburgh, Pennsylvania, United States

[7] Walther Straub Institute of Pharmacology and Toxicology, Ludwig-Maximilians-University Munich, Munich, Germany; Member of the German Center for Lung Research (DZL/CPC-M), Munich, Germany

* Correspondence: heiko.adler@lrz.uni-muenchen.de; Phone: +49 89 2180-73842

Supplementary Figures

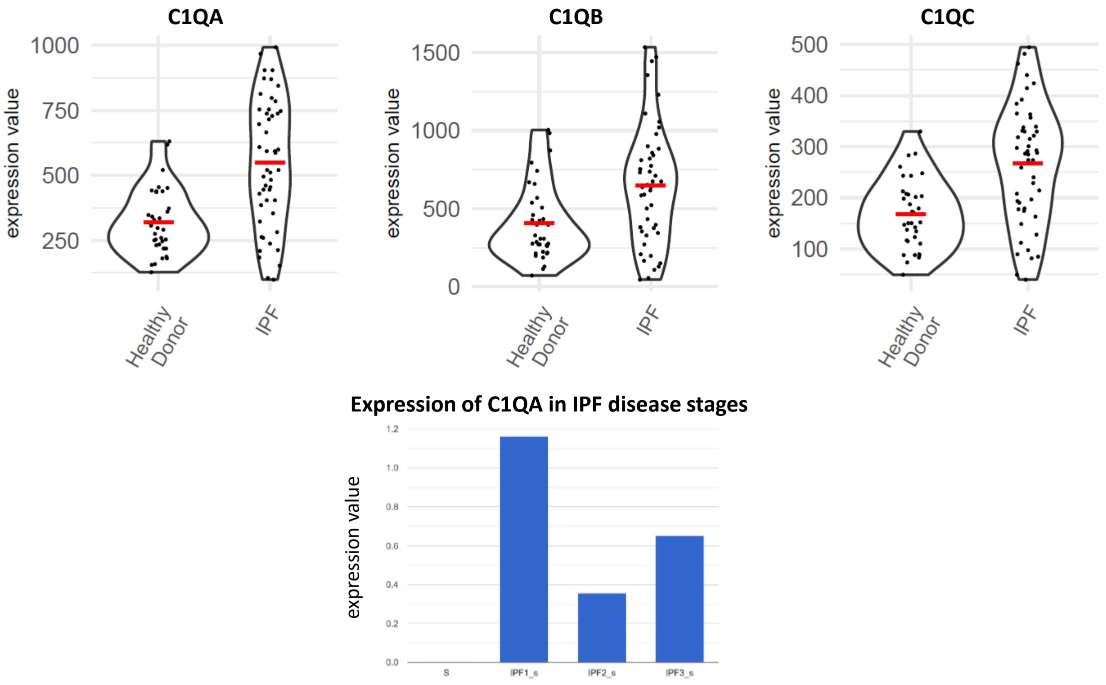

Supplementary Figure S1: C1q mRNA expression is upregulated in IPF patients. A)-C) Raw data were obtained from GSE124685 [GEO Accession viewer GSE124685; 02/05/2020. Available from: https://www.ncbi.nlm.nih.gov/geo/query/acc.cgi?acc=GSE124685 (Accessed: 02/05/2020)], analyzed with R, and displayed as violin plots. Each dot represents one individual. Red bars indicate the mean. D) A plot was generated from GSE124685 [GEO Accession viewer GSE124685; 02/05/2020. Available from: https://www.ncbi.nlm.nih.gov/geo/query/acc.cgi?acc=GSE124685 (Accessed: 02/05/2020)] with an online tool available at http://sb.cs.cmu.edu/IPFReg/ (Accessed: 24/02/2021). "S" indicates the control group, "IPF1_s" refers to early-stage, "IPF2_s" to progressive-stage, and "IPF3_s" to endstage disease [1].

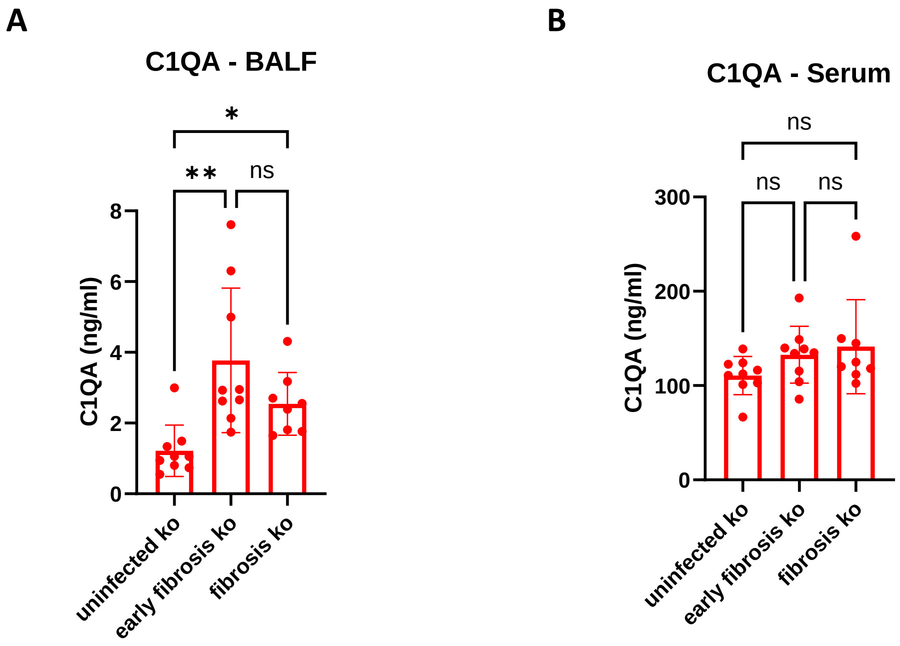

Supplementary Figure S2: Increased levels of C1QA in BAL fluids but not in serum of fibrotic mice. BALFs (A) and sera (B) of uninfected and MHV-68 infected IFN-γ R−/− (KO) mice were collected during early fibrosis and fibrosis. C1QA concentration was measured by ELISA. Each symbol represents an individual mouse, and the columns represent the mean ± SD. The asterisks indicate a statistically significant difference (Kruskal-Wallis test). ns: not significant.

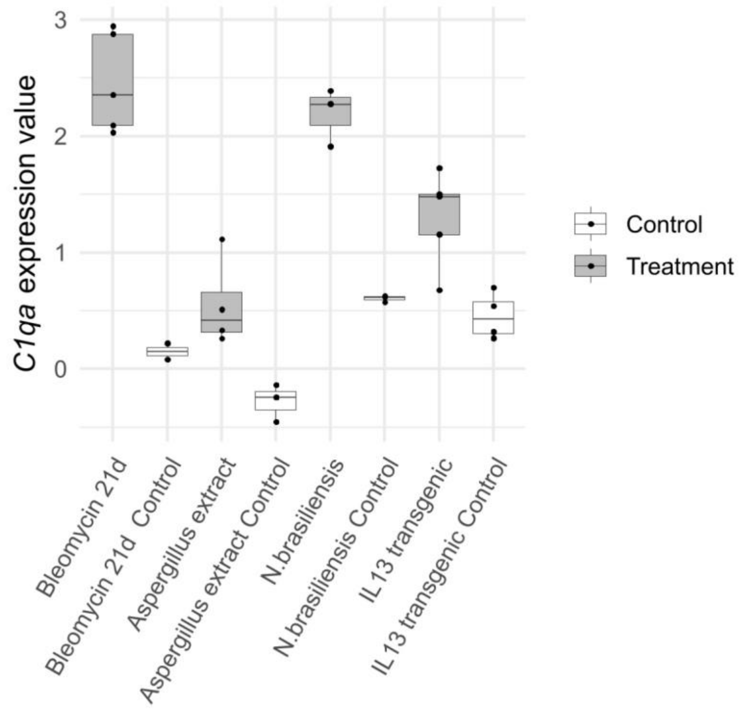

Supplementary Figure S3: Higher C1qa gene expression in fibrotic mice confirmed in various models in a GEO data set. Raw data was obtained from GSE4231 and analyzed with R. Each dot represents data from one individual sample. The median is shown, box limits represent the 25th and 75th percentiles, and whiskers reach to largest value with a maximum length of 1.5 * inter-quartile range from the hinge.

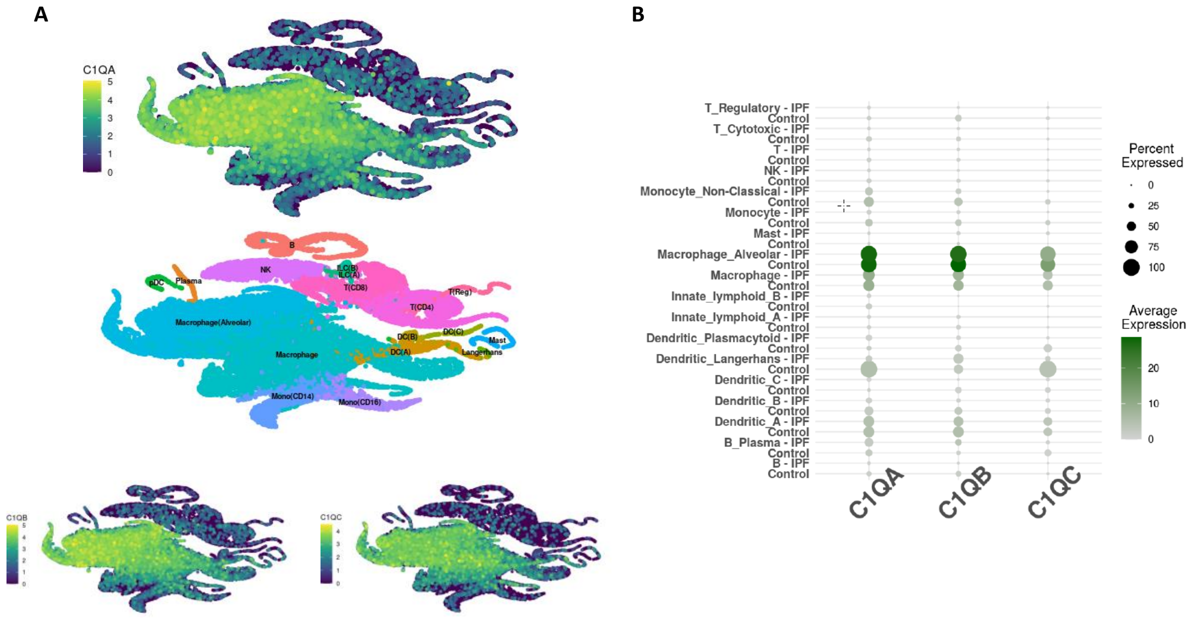

Supplementary Figure S4: C1q gene expression in single cell RNA sequencing data. A) UMAP Explorer. B) Batch Explorer. The visualizations of the single cell sequencing show that in the human lung, macrophages are the main producers of all three C1q subcomponents (C1QA, C1QB, C1QC). The images were generated with the online tool available at http://www.ipfcellatlas.com from the GSE136831 dataset (Kaminski/Rosas) (Accessed: 26.01.2024).

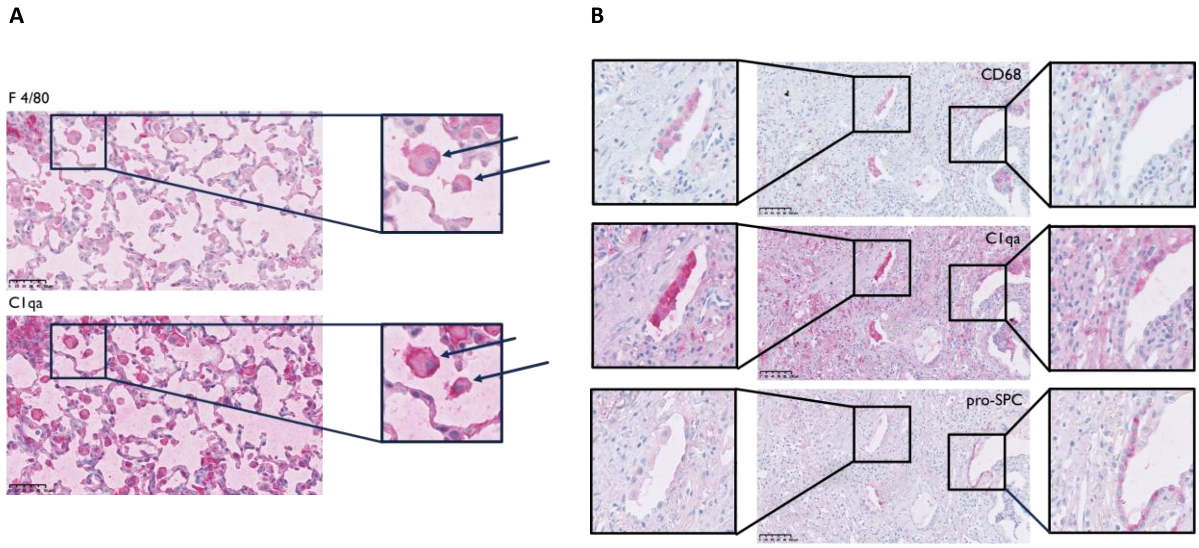

Supplementary Figure S5: C1QA is expressed by macrophages in both mouse and human. A) By means of IHC, sequential slices of murine lung tissue show co-localization of the macrophage marker F4/80 with C1QA. Sequential slices of lung tissue of uninfected and MHV-68 infected C57BL/6 and IFN-γ $R^{-/-}$ mice were immuno-stained for C1QA and F4/80 and counterstained with haemalaun solution. Representative images from a fibrotic mouse lung are shown. Scale bar indicates 50 μm. B) Sequential slices of human lung tissue show co-localization of the macrophage marker CD68 with C1QA. Sequential slices of lung tissue of IPF patients and donors were immuno-stained for C1QA, CD68 and proSPC and counterstained with haemalaun solution. Representative images from an IPF lung are shown. Scale bar indicates 100 μm.

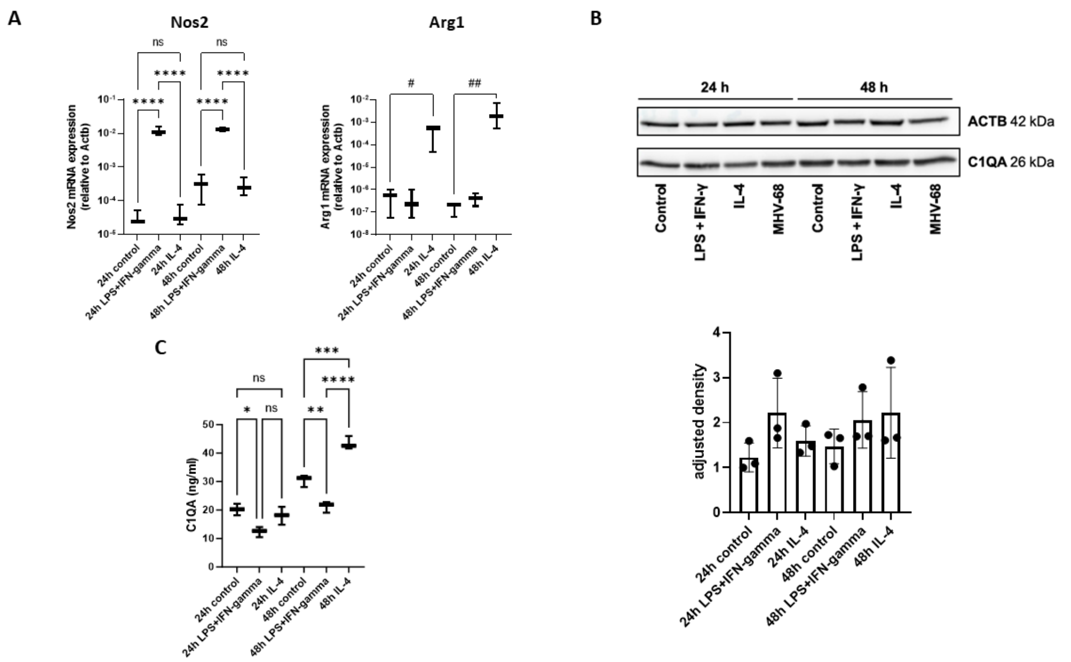

Supplementary Figure S6: Macrophages polarized to M2 secrete higher amounts of C1q. A) to C) MH-S cells were treated with medium, LPS + IFN-γ or IL-4, and cells and supernatants were harvested after 24 h and 48 h. A) Gene expression was analyzed by qRT-PCR, normalized to β-actin (Actb), and displayed relative to Actb. Results are from 3 independent experiments and shown as Box-Whisker-Plots. The asterisks indicate a statistically significant difference (One-way ANOVA). ns: not significant. B) C1QA and β-actin (ACTB) protein levels from cell lysates were analyzed by Western blot (upper panel). Band density was measured with ImageJ, adjusted to β-actin levels, and displayed as adjusted density (lower panel). Each symbol represents an individual sample, and the columns represent the mean ± SD. C) C1QA concentration in supernatants was measured by ELISA. Results are from 3 independent experiments and shown as Box-Whisker-Plots. The asterisks indicate a statistically significant difference (One-way ANOVA). ns: not significant; #: not significant due to high variation; ##: p = 0,1408.

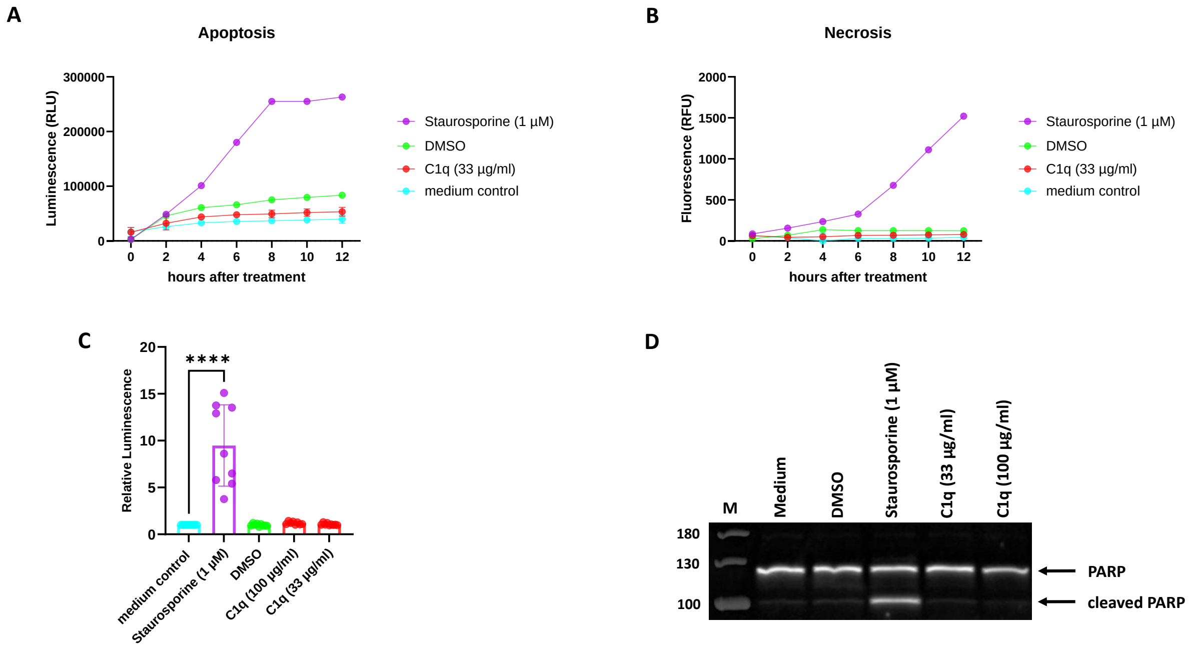

Supplementary Figure S7: C1q induced cell death of alveolar epithelial cells is neither apoptosis nor necrosis. MLE-12 cells were treated with C1q, culture medium or DMSO as negative controls and Staurosporine as positive control. A) Apoptosis and B) necrosis were determined using the RealTime-Glo™ Annexin V Apoptosis and Necrosis Assay from Promega. Luminescence and fluorescence were measured multiple times from 0 h to 12 h. Medium blank values were deducted. n=1 for DMSO and Staurosporine; n=3 (means $\pm$ SD) for medium and C1q. C) Apoptosis was additionally determined 6 h after treatment using the Caspase-Glo® 3/7 Assay from Promega and D) by Western blot for cleaved PARP. In panel C), each symbol represents an individual experiment, and the columns represent the mean $\pm$ SD. The asterisks indicate a statistically significant difference (One-way ANOVA).

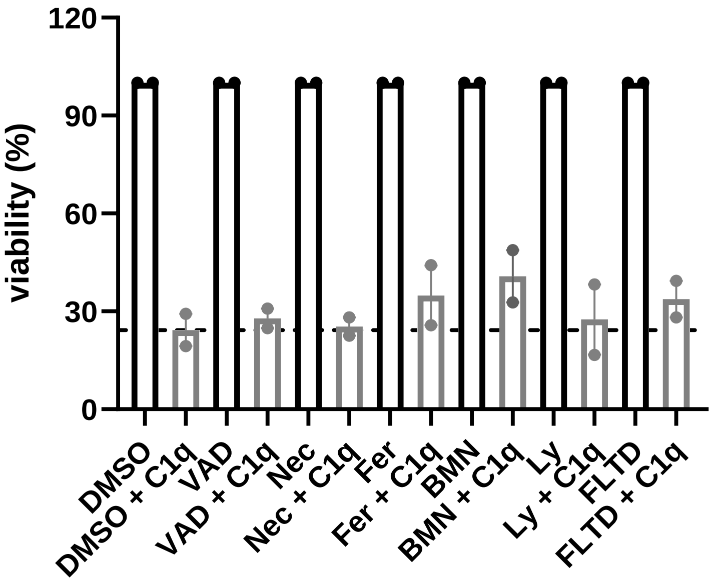

Supplementary Figure S8: C1q induced cell death of alveolar epithelial cells is not apoptosis, pyroptosis, necroptosis, parthanatos, ferroptosis or autophagy-dependent cell death. MLE-12 cells were treated with DMSO as negative control or with C1q in the presence or absence of inhibitors of specific cell death pathways. Cell viability was determined by MTT assay. Viability of the cells after treatment with the respective inhibitor alone was always set to 100 percent. The dashed line indicates cell viability after treatment with C1q (100 µg/ml) in the absence of any inhibitor. Each symbol represents an individual experiment, performed in duplicates, and the columns represent the mean ± SEM. VAD: Z-VAD-FMK (apoptosis inhibitor, 20 µM); Nec: Necrostatin-1 (necroptosis inhibitor, 20 µM); Fer: Ferrostatin-1 (ferroptosis inhibitor, 10 µM); BMN: BMN-673 (parthanatos inhibitor, 10 µM); Ly: Ly294002 (autophagy inhibitor, 10 µM); FLTD: Ac-FLTD-CMK (pyroptosis inhibitor, 10 µM).

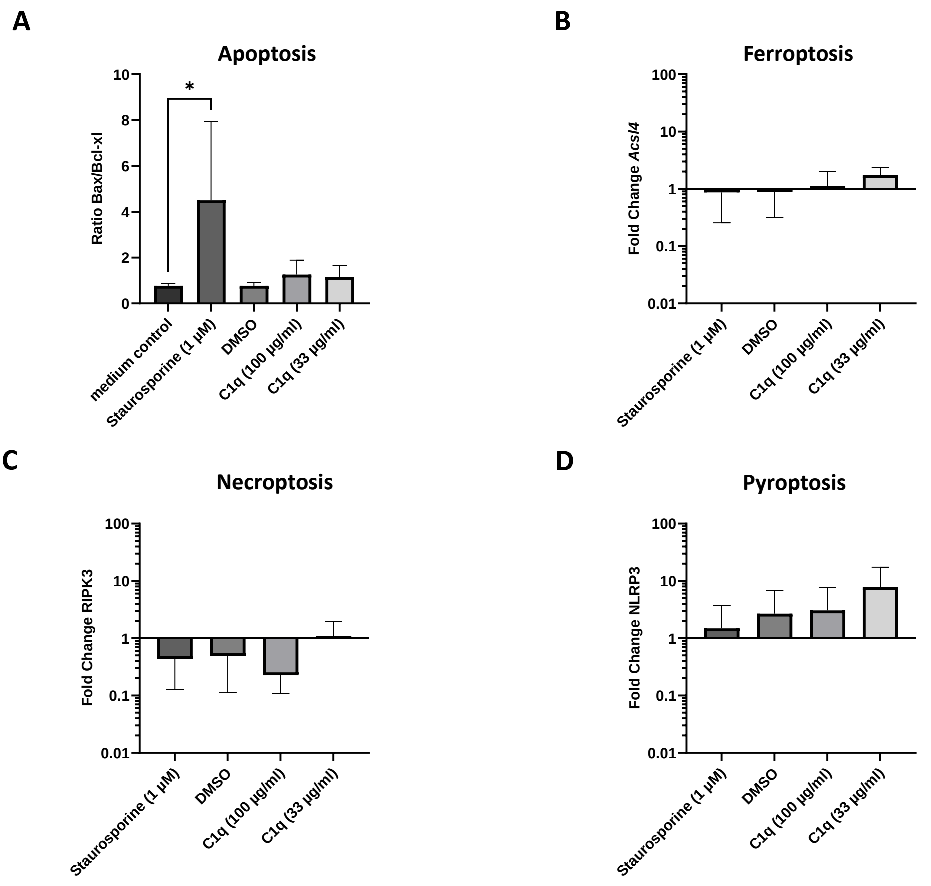

Supplementary Figure S9: Gene expression of characteristic cell death-associated genes in MLE-12 cells after treatment with C1q and controls. Gene expression was analyzed by qRT-PCR, normalized to Actb and displayed as fold change relative to the medium control (which was set to 1), except for panel A) where the ratio of Bax to Bcl-xl expression, indicative of apoptosis, is displayed (n=4 independent experiments). B) Expression of Acsl4, indicative of ferroptosis (n=3 independent experiments). C) Expression of RIPK3, indicative of necroptosis (n=3 independent experiments). D) Expression of NLRP3, indicative of pyroptosis (n=3 independent experiments). Data shown are means + SD. The asterisk indicates a statistically significant difference, all other values are not significantly different compared to the medium control (which is set to 1) (One-way ANOVA).

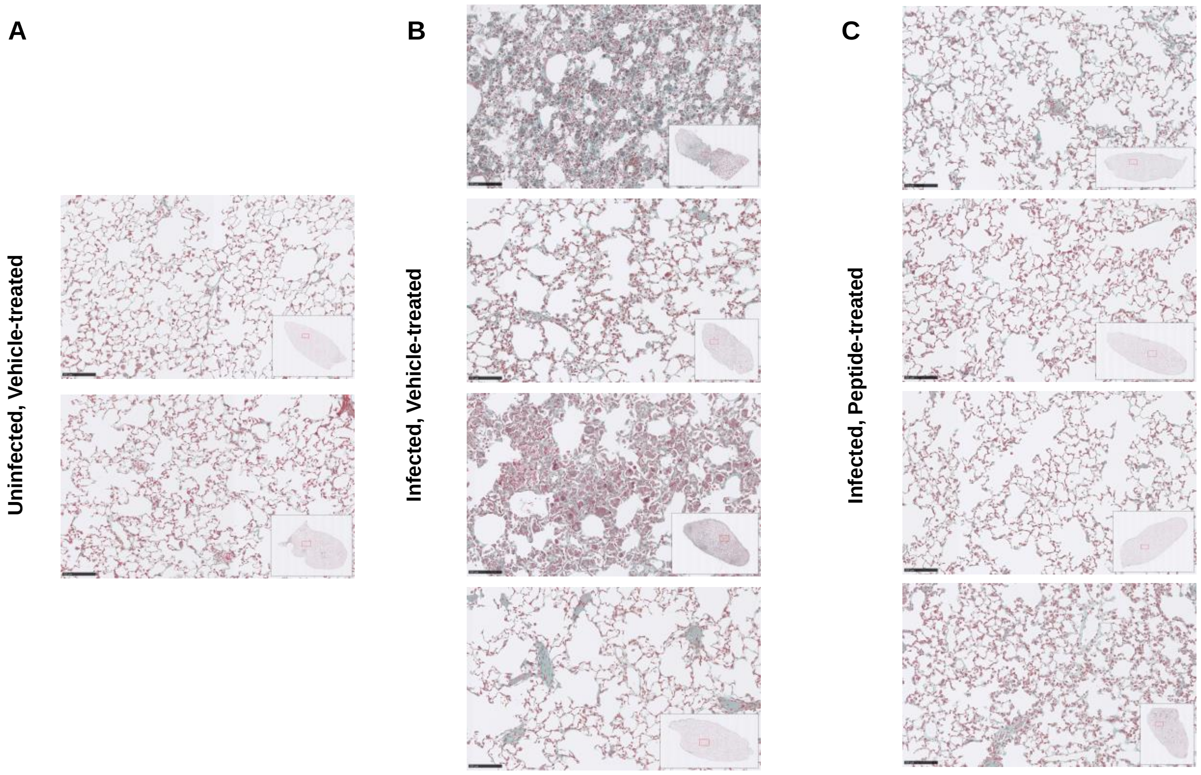

Supplementary Figure S10: High resolution images of the Masson-Goldner (MG) trichrome-stained sections shown in Figure 3B. Magnification: 20x. Scale bars indicate 100 µm.

Supplementary Materials and Methods

In vivo experiments

C57BL/6 mice were purchased from Charles River Laboratories (Sulzfeld, Germany). IFN-γ-R-/- mice on C57BL/6 background were originally obtained from the Jackson Laboratory (Bar Harbor, ME, USA) and subsequently bred and propagated under SPF conditions at the Helmholtz Zentrum München. Mice were housed in individually ventilated cages during the murine gammaherpesvirus 68 (MHV-68) infection period. Mice (8-12 weeks old) were infected intranasally (i.n.) with $5x10^4$ plaque forming units of MHV-68 diluted in phosphate-buffered saline (PBS) in a total volume of 30 µl. Prior to i.n. infection, mice were anesthetized with ketamine-xylazine or with medetomidine-midazolam-fentanyl. To inhibit C1q in vivo, mice were either treated intraperitoneally (i.p.) with 2J peptide [2] (2 mg/kg) or with vehicle control twice a week as described by others [3]. Blood oxygen saturation (SpO_2) was determined using a Pulse Oximeter for mice (MSTAT-JR, Kent Scientific Corporation). At the time points indicated in the respective figures, mice were sacrificed by cervical dislocation. Subsequently, BAL was performed and lung tissues were processed for the following experiments: the left lobe was inflated and fixed in 4% buffered formalin for histological and immunohistochemical examination, and the remaining lobes were stored at -80° C and used for RNA isolation for qRT-PCR to determine gene expression or for preparation of whole lung tissue protein extracts and Western blot analysis. All animal experiments were in compliance with the German Animal Welfare Act (German Federal Law §8 Abs. 1 TierSchG), and the protocols were approved by the local Animal Care and Use Committee (District Government of Upper Bavaria; permit numbers 124-08, 154-13 and 18-97).

BAL and preparation of tissue for histopathology

BAL and tissue preparation for histopathology were done as previously described [4].

Cell Culture

MH-S cells (ATCC CRL-2019) were cultured in RPMI 1640 (Gibco, Darmstadt, Germany) supplemented with 10% heat-inactivated fetal calf serum (FCS; PAN Biotech, Aidenbach, Germany), 2 mM L-glutamine, 100 U/ml Penicillin, 100 µg/ml Streptomycin and 0.1% beta-mercaptoethanol. MLE-12 cells (ATCC CRL-2110) were cultured in DMEM (Gibco) supplemented with 10% heat-inactivated FCS (PAN Biotech), 2 mM L-glutamine, 100 U/ml Penicillin and 100 µg/ml Streptomycin.

MTT assay

To determine cell viability, MTT assays were carried out. $3x10^3$ or $1x10^4$ MLE-12 cells per well were seeded in 96-well plates in 200 µl of culture medium and were grown overnight. Then, the cells were treated with C1q (Merck Millipore). In experiments in which inhibitors of specific cell death pathways were included, the inhibitors were applied 2 h prior to treatment with C1q, as described by others [5]. After C1q was added, the cells were incubated at 37° C, 5% CO_2 for 45 or 69 h before the MTT solution was added to a final concentration of 1 mg/ml. The cells were incubated at 37° C, 5% CO_2 for another 3 h prior to discarding the medium and adding 100 µl of dissolving solution to each well. The plate was shaken for approx. 15 min until the content was homogenized. Then, the optical density was measured with a TECAN© Sunrise™ ELISA reader at 590 nm wavelength.

Cytotoxicity Detection - LDH-Assay

To investigate the potential cytotoxic effects of C1q, MLE-12 cells were seeded at a density of $5x10^4$ per well in 1 ml culture medium in a 24-well plate, and next day treated with C1q. Treatment with culture medium only served as a negative control and treatment with Triton X 100 (1%) served as a positive control. The supernatants were collected 48 h after treatment and stored at -80° C until the LDH assay was carried out with the Roche Cytotoxicity Detection Kit according to the manufacturer's instructions.

Apoptosis and Necrosis Assay

To investigate whether C1q induces cell death and to discriminate between apoptosis and necrosis, an Apoptosis and Necrosis Assay (RealTime-Glo™Annexin V, Promega) was carried out. MLE-12 cells were seeded at a density of $5x10^3$ per well in 50 µl of culture medium in a 96-well plate. Next day, cells were treated with C1q as described for cytotoxicity detection, and 2x Detection Solution was added to each well according to the manufacturer's instructions. Measurements of luminescence and fluorescence were performed at 0 h, 4 h, 6 h, 8 h, 10 h, 14 h and 24 h after stimulation using the GloMax Discover 3000 RealTime-Glo Annexin V protocol.

Apoptosis Assay

Apoptosis was determined using the Caspase-Glo® 3/7 Assay Kit from Promega according to the instructions of the manufacturer. On the day before the measurement, $1x10^4$ MLE-12 cells per well were seeded in a white 96-well plate (Nunc™ MicroWell™ 96-Well, Thermo Fisher Scientific). After 18 to 24 hours, the cells were treated with either C1q (100 µg/ml and 33 µg/ml), medium and DMSO as negative controls, and 1µM Staurosporine (Cell Signaling Technologies) as positive control. The cells were incubated at 37° C for 6 h, and then 100 µl of Caspase-Glo Reagent were added to each well. The plate was then shaken at 300 rpm for 30 s, followed by one hour incubation at room temperature. Subsequently, luminescence (RLU) was measured with the GloMax® Discover Microplate Reader (Promega) using a standard protocol.

Multiparametric monitoring of cellular metabolic responses

To continuously measure oxygen consumption rate (OCR) and extracellular acidification rate (ECAR), and to perform in parallel periodic microscopic imaging, the CYRIS® FLOX analysis platform (INCYTON® GmbH, Planegg, Germany) was utilized [6]. MLE-12 cells cultured as described above were detached, counted, and seeded at a density of $2x10^4$ cells per well (47

mm² culture area) in replicates in a special sterile sensor plate. Four wells were filled with pure culture medium to serve as cell-free control. The sensor plate was then incubated for 4 h under standard conditions in a cell culture incubator to allow the cells to adhere. Meanwhile, the CYRIS® FLOX analysis platform was set up for the assay. For this purpose, deep well plates with C1q (33 µg/ml for treatment) in standard measurement medium (DMEM high glucose with 4 mM L-glutamine and without sodium pyruvate, $NaHCO_3$ and phenol red supplemented with 10 % heat-inactivated FCS) or without C1q (for initial measurement prior to treatment and for control treatment) were prepared and placed in the CYRIS® FLOX climate chamber (37° C, 90 % relative humidity, 21 % O_2). After sufficient adhesion of the cells in the sensor plate, the culture medium on the cells was replaced by fresh measurement medium, the special sensor plate lid was put on, the sensor plate was inserted into the platform and the assay was started. The assay setting included a prior treatment measurement period without C1q for 24 h (baseline) and then treatment of the cells with C1q (33 µg/ml) for 48 h. Untreated control cells were given only measurement medium. The assay was performed automatically, and the acquired data were displayed in realtime. Throughout the assay, OCR and ECAR of all wells were recorded, as well as the morphology based on microscope images at intervals of 20 min. After the assay, the data were analyzed as follows: Metabolic rates were first normalized, i.e. each rate was divided by the reference rate at the time of C1q application. Then, for each individual group (untreated control or treatment with C1q), the mean values of the metabolic rates for ECAR and OCR and the respective standard deviations were calculated.

Macrophage Polarization

MH-S macrophages were polarized towards the M1 phenotype by classical activation with interferon gamma (IFN-γ) (20 ng/ml) and lipopolysaccharide (LPS) (100 ng/ml), and towards the M2 phenotype by alternative activation with interleukin-4 (IL-4) (20 ng/ml). Culture medium served as negative control. For RNA isolation, MH-S cells were seeded at a density

of $5x10^4$ per well in 1 ml of culture medium in a 24-well plate, treated next day, and harvested 48 h after treatment. For protein isolation, MH-S cells were seeded at a density of $2x10^5$ per well in 2 ml of culture medium in 6-well plates, and harvested 24 h or 48 h after treatment. The supernatants were collected for ELISA analysis.

RNA Isolation

Whole lung tissue (WLT) was prepared for RNA isolation by mechanical disruption according to the manufacturer's instructions of the Macherey-Nagel NucleoSpin® RNA Plus RNA isolation kit. For RNA isolation from cultured cells, $5x10^4$ cells per well were seeded in 1 ml medium in 24-well plates and harvested 24 or 48 h after treatment. The RNA was isolated using the Macherey-Nagel NucleoSpin® RNA Plus RNA isolation kit according to the manufacturer's instructions.

Quantitative Reverse Transcription Polymerase Chain Reaction (qRT-PCR)

500 ng RNA was reverse-transcribed using the High Capacity cDNA Reverse Transcription Kit (Applied Biosystems, Foster City, CA, USA) according to the instructions of the manufacturer, or subjected to mock reverse-transcription in the absence of the enzyme (-RT control). Subsequently, 2 µl of the resulting cDNA (or double-distilled water as non-template control) were used as template for PCR amplification with the ABI 7300 Real Time PCR System, using Power SYBR Green PCR Master Mix and universal cycling conditions (Applied Biosystems). The primers which were used are shown in Supplementary Table 1.

Protein Isolation

Whole lung tissue (WLT) samples were kept on ice at all times. The samples were homogenized in RIPA-buffer (NaCl [5 M], TRIS [1 M, pH 7.2], SDS [10%], Triton X100 [1%], EDTA [0.5 M], Desoxychlorat [10%]) using a FastPrep-24 instrument (MP Biomedicals, Heidelberg, Germany). The homogenized samples were transferred into fresh tubes, kept on ice for 30 min and vortexed repeatedly. After 30 min, the samples were centrifuged, and the

supernatant was carefully transferred into a fresh tube and stored at -80° C until further use. Cultured cells were lysed in RIPA-buffer and stored at -80° C until further use. Protein concentrations were measured by Bradford assay with Coomassie protein assay reagent (Thermo Scientific) according to manufacturer's instructions.

Western Blot analysis

Samples were subjected to SDS-PAGE and transferred to nitrocellulose membranes. Signals were detected by measuring luminescence using a Fusion Imager (Vilber Lourmat, Germany) and saved as an image. If applicable, protein quantification was carried out with ImageJ. Antibodies used for Western Blot were as follows: rabbit anti-C1QA (Biorbyt), peroxidase-conjugated mouse anti-β-actin (Sigma-Aldrich), rabbit anti-PARP (Cell Signaling Technologies) and peroxidase-conjugated donkey anti-rabbit (GE Healthcare).

Enzyme-Linked Immunosorbent Assay (ELISA)

To investigate C1q secretion, C1qA concentrations in murine and human BAL fluids as well as in supernatants of macrophage cell cultures were measured with ELISA using the Biobyrt Human and Mouse C1qA ELISA Kits according to the manufacturer's instructions.

Histopathological examination

To verify fibrotic changes in lung tissue, murine and human lung tissue sections were stained with hematoxylin and eosin (HE) for routine histologic examination and Masson trichrome staining to delineate collagen according to standard procedures as described previously [7]. All slides were scanned with the NanoZoomer Slide Scanner. For quantitative analysis, HE-stained sections were analyzed using ImageJ. Morphometric analyses were performed on HE-stained sections by determining mean septal thickness and alveolar diameter. AxioVision software (Carl Zeiss MicroImaging GmbH, Jena Germany) was used for these analyses as described previously [8].

Immunohistochemistry (IHC)

Antibody staining was performed in subsequent slides to examine the location of C1qA in murine and human lung tissue. Staining was carried out according to standard procedures as previously described [9] using the ZytoChem-Plus AP Kit (Zytomed Systems). All slides were scanned with the NanoZoomer Slide Scanner. The antibodies used were as follows: rabbit anti-C1QA human (Biorbyt), rabbit anti-C1QA mouse (Biorbyt), rat anti-F4/80 mouse (Bio-Rad), mouse anti-CD-68 human (Abcam), rabbit anti-proSP-C human (Millipore) and rabbit anti-CD206 human (Abcam).

Analysis of Publicly Accessible Datasets

If available, microarray datasets published in the Gene Expression Omnibus (GEO) were analyzed with the tools provided by the publishers. Otherwise, the datasets were analyzed by standard statistical methods with R version 3.6.0.

Statistical analysis

Datasets were analyzed using the GraphPad Prism software, vs9 (GraphPad Software, Inc., San Diego, CA, USA) as indicated in the figure legends. P-values were expressed as follows: $0.05 > p > 0.01$ as *; $0.01 > p > 0.001$ as **; and $p < 0.001$ as ***. Results with a p-value < 0.05 were considered significant.

Supplementary Table 1: Primers used for qRT-PCR

**Primer**	**Sequence (5’ - 3’)**
mC1qa.for	AAAGGCAATCCAGGCAATATC
mC1qa.rev	TGGTTCTGGTATGGACTCTCC
mb-actin.for	TCCATCATGAAGTGTGACGT
mb-actin.rev	GAGCAATGATCTTGATCTTCAT
mNos2.for	CCTGTGAGACCTTTGATG
mNos2.rev	CCTATATTGCTGTGGCTC
mArg1.for	GGAACCCAGAGAGAGCATGA
mArg1.rev	TTTTTCCAGCAGACCAGCTT
mAcsl4.for	ACTTACCTTTGGCTCATG
mAcsl4.rev	CAGTACAGTACAATCACCCT
mRIPK3.for	GAAGACACGGCACTCCTTGGTA
mRIPK3.rev	CTTGAGGCAGTAGTTCTTGGTGG
mNLRP3.for	TCACAACTCGCCCAAGGAGGAA
mNLRP3.rev	AAGAGACCACGGCAGAAGCTAG
mBim.for	GCCTGCTTTGCTCTCTCCAT
mBim.rev	CCTACACGTGTGCCGTTTGT
mBcl-xl.for	AGACCCCCAGTGCCATCAAT
mBcl-xl.rev	CCCGCCAAAGGAGAAAAA
mBax.for	CCAGCTCTGAACAGATCATG
mBax.rev	AGCTCCATATTGCTATCCAG

Extended introduction to the topic of the study

Idiopathic pulmonary fibrosis (IPF) is a devastating interstitial lung disease (ILD) with a median survival time in untreated patients of only 2-5 years after diagnosis due to respiratory failure [10-12]. IPF is characterized by damage of alveolar epithelial cells, excessive deposition of extracellular matrix (ECM) and enhanced activation and proliferation of fibroblasts, altogether eventually leading to the disruption of normal lung architecture and loss of lung function [13]. The underlying cause of IPF is not yet known [14], but it is assumed that the interplay of genetic (mutations and polymorphisms) and environmental factors ultimately lead to IPF [13]. Viral infections, for example with herpesviruses, have been proposed as environmental factors driving the susceptibility for IPF [15]. More than 20% of previous severe acute respiratory syndrome (SARS) survivors had pulmonary fibrosis one year later, indicating that pulmonary fibrosis is a long-term problem [16,17]. First reports from COVID-19 patients described computed tomography (CT) patterns related to lung fibrosis, and it was speculated that the issue of fibrosis will emerge in the future [18]. Indeed, although the COVID-19 pandemic as such is over, it is now expected that post-COVID lung fibrosis might become a huge medical and public health issue (European Respiratory Society: ERS Vision Live on "An update on post-COVID lung fibrosis"; 04.12.2023). Currently, there are two FDA approved drugs for the treatment of IPF patients: Ofev (nintedanib) and Esbriet (pirfenidone) [19-21]. These drugs slow down disease progression but do not block or even reverse the course of the disease. In addition, they have adverse effects. Thus, currently, the only curative therapy remains organ transplantation, and new drugs with fewer adverse effects are highly desirable [22].

The complement system is part of the innate immune system, initially considered as a defense against infections [23]. However, it quickly became clear that it has multiple important additional functions including the orchestration of immunological and inflammatory processes, thereby significantly contributing to cell homeostasis [24]. Due to its important homeostatic role, too

much or too little complement activation can lead to the development of disease [25,26]. For example, complement activation is implicated in the pathogenesis of progressive tubulointerstitial renal fibrosis [27]. The complement system has also been suggested to be involved in the development of various lung diseases, and very early studies generated some evidence of complement activation in IPF [28]. However, the specific contributions of complement to the pathogenesis of IPF remain elusive. A better understanding of the potential mechanisms of complement-mediated injury in IPF might therefore help to identify new targets for therapeutic interventions [28].

Very recently, bioinformatic analyses identified high levels of the complement component C1q in pulmonary fibrosis and recognized C1q as a potential diagnostic and prognostic marker [29]. In a mouse model, C1q has been shown to exacerbate silica-induced pulmonary fibrosis [30]. C1q, together with C1r and C1s, forms the complement component C1 that constitutes the activator of the classical complement pathway [25]. In addition to its role in the complement cascade, C1q has a variety of other functions [31]. For example, C1q was found to interact with signaling pathways that are also dysregulated in IPF such as the Wnt-signaling and β-catenin signaling pathways, and to promote an aging-associated decline in tissue regeneration [32,33]. Furthermore, C1q was found to promote polarization of macrophages towards the M2 phenotype [34,35] and to be a macrophage chemoattractant [36]. Many cells have been reported to produce C1q, in particular those of the monocyte/macrophage lineage, which may result in substantial local concentrations of C1q in tissues or organs [25].

In this study, we aimed to explore whether C1q might play a role in IPF and if yes, to dissect underlying mechanisms in order to identify potential new targets for therapeutic intervention.

Extended discussion of the findings of the study

C1q has been suggested as a potential diagnostic and prognostic marker for IPF [29] and was

found to interact with signaling pathways that are dysregulated in IPF [32]. It can attract fibroblasts to sites of injury and can mediate adhesion of fibroblasts to extracellular matrix proteins [31]. Therefore, in this study, we investigated whether C1q might play a role in the pathogenesis of IPF. We found increased levels of C1qA in BAL fluids of both IPF patients and fibrotic mice that were produced by macrophages, with M2 macrophages secreting higher amounts of C1qA than M1 macrophages. Our finding that C1q was produced by macrophages is in line with previous reports and supports the notion that this may lead to high local tissue or organ concentrations [25]. Along this line, C1qA has recently been proposed as part of the transcriptomic signature that distinguishes disease-associated monocyte-derived macrophages from their tissue-resident counterparts [37]. Since C1q can promote polarization of macrophages to the M2 phenotype [34,35] and can function as a chemoattractant for macrophages [36], it seems to promote its own local production in a positive feedback loop. This might be particularly relevant since pro-fibrotic M2 macrophages play an important role in the development of IPF, for example by production of pro-fibrotic factors, by orchestrating local inflammatory reactions and by enhancing the survival and activation of myofibroblasts (reviewed in [38-41]). Thus, reprogramming of pro-fibrotic macrophages has already been suggested as an attractive option for the treatment of pulmonary fibrosis [42].

The fibrotic response in IPF is driven primarily by injury and dysfunction of AECII [13,14,43]. For example, widespread epithelial cell apoptosis has been reported in IPF [13]. Surprisingly, in our study, C1q did not induce AECII cell death since the reduction in metabolic activity - as determined by MTT-assay - could not be prevented by standard inhibitors of apoptosis, pyroptosis, necroptosis, parthanatos, ferroptosis or autophagy-dependent cell death. In contrast, in prostate and ovarian cancer cells, C1q has been shown to induce apoptosis [44,45]. Instead, by realtime monitoring of cellular metabolic responses (OCR and ECAR) and simultaneous microscopic imaging, we showed here that C1q disturbes the metabolism of AECII, thus

suggesting a new mode of contribution to fibrosis development. It has been described that metabolic reprogramming and dysregulation are typical features of IPF lungs [46]. The metabolic decline in AECII induced by macrophage-derived C1q might contribute in a variety of ways to fibrosis development: Since AECII have high metabolic demands during lung injury and repair [47], an intact AECII metabolism is required for alveolar epithelial regeneration during lung injury [48]. Abnormalities in AECII metabolism may result in reduced AECII self-renewal [49] and in disturbed proliferation and differentiation [50]. Dysfunctional lung epithelial cells may produce pro-fibrotic factors including transforming growth factor β and matrix metalloproteinases [41]. Our findings further support the notion that macrophage - epithelial cross-talk is an important process both in health and disease [51-54].

We demonstrated that inhibition of C1q in vivo reduces fibrosis development and counteracts a drop in peripheral oxygen saturation, one of the most important clinical progression parameters directly related to the quality of life of patients, thus identifying C1q as a target molecule for therapeutic intervention. Inhibition of complement components is an attractive strategy for the treatment of various diseases [25]. Inhibition of C1q has already been suggested as a potential therapy to prevent arteriosclerosis in patients with hypertension [33] and carcinogenesis in patients with chronic hepatitis [3] or to treat pulmonary fibrosis [30].

In summary, we identified C1q, produced by macrophages, as an inducer of a metabolic disorder of AECII, emphasizing the importance of macrophage - epithelial cross-talk for the development of pulmonary fibrosis, and propose C1q as a potential new therapeutic target.

Supplementary references

1 McDonough, J. E. *et al.* Transcriptional regulatory model of fibrosis progression in the human lung. *JCI. Insight* **4**, e131597 (2019).

2 Roos, A. *et al.* Specific inhibition of the classical complement pathway by C1q-binding peptides. *J. Immunol* **167**, 7052-7059 (2001).

3 Ho, T. C. *et al.* Complement C1q mediates the expansion of periportal hepatic progenitor cells in senescence-associated inflammatory liver. *Proc. Natl. Acad. Sci. U. S. A* **117**, 6717-6725 (2020).

4 Zhang, W. *et al.* S100a4 Is Secreted by Alternatively Activated Alveolar Macrophages and Promotes Activation of Lung Fibroblasts in Pulmonary Fibrosis. *Front Immunol* **9:1216.** doi: 10.3389/fimmu.2018.01216 (2018).

5 Zhang, L., Jiang, X., Pfau, D., Ling, Y. & Nathan, C. F. Type I interferon signaling mediates Mycobacterium tuberculosis-induced macrophage death. *J. Exp. Med* **218**, e20200887 (2021).

6 Heichler, C., Nagy, M. & Wolf, P. Evaluation of hepatotoxic effects of acetaminophen on HepG2 cells by parallel real-time monitoring in a multi-sensor analysis platform for automated cell-based assays. *Bioenergetics Communications* **2022.6**, doi: 10.26124/bec:2022-0006 (2022).

7 Mahavadi, P. *et al.* Epithelial stress and apoptosis underlie Hermansky-Pudlak syndrome-associated interstitial pneumonia. *Am J Respir Crit Care Med* **182**, 207-219, doi:10.1164/rccm.200909-1414OC (2010).

8 Wasnick, R. *et al.* Notch1 Induces Defective Epithelial Surfactant Processing and Pulmonary Fibrosis. *Am J Respir Crit Care Med* **207**, 283-299, doi:10.1164/rccm.202105-1284OC (2023).

9 Korfei, M. *et al.* Aberrant expression and activity of histone deacetylases in sporadic idiopathic pulmonary fibrosis. *Thorax* **70**, 1022-1032, doi:10.1136/thoraxjnl-2014-206411 (2015).

10 Maher, T. M., Wells, A. U. & Laurent, G. J. Idiopathic pulmonary fibrosis: multiple causes and multiple mechanisms? *Eur. Respir. J* **30**, 835-839 (2007).

11 Collard, H. R. *et al.* Acute exacerbations of idiopathic pulmonary fibrosis. *Am. J. Respir. Crit Care Med* **176**, 636-643 (2007).

12 Raghu, G. *et al.* Idiopathic pulmonary fibrosis in US Medicare beneficiaries aged 65 years and older: incidence, prevalence, and survival, 2001-11. *Lancet Respir Med* **2**, 566-572, doi:10.1016/s2213-2600(14)70101-8 (2014).

13 King, T. E., Jr., Pardo, A. & Selman, M. Idiopathic pulmonary fibrosis. *Lancet* **378**, 1949-1961 (2011).

14 Fernandez, I. E. & Eickelberg, O. New cellular and molecular mechanisms of lung injury and fibrosis in idiopathic pulmonary fibrosis. *Lancet* **380**, 680-688 (2012).

15 Vannella, K. M. & Moore, B. B. Viruses as co-factors for the initiation or exacerbation of lung fibrosis. *Fibrogenesis. Tissue Repair* **1**, 2 (2008).

16 Hui, D. S. *et al.* The 1-year impact of severe acute respiratory syndrome on pulmonary function, exercise capacity, and quality of life in a cohort of survivors. *Chest* **128**, 2247-2261, doi:10.1378/chest.128.4.2247 (2005).

17 Xie, L. *et al.* Dynamic changes of serum SARS-coronavirus IgG, pulmonary function and radiography in patients recovering from SARS after hospital discharge. *Respir Res* **6**, 5, doi:10.1186/1465-9921-6-5 (2005).

18 Ye, Z., Zhang, Y., Wang, Y., Huang, Z. & Song, B. Chest CT manifestations of new coronavirus disease 2019 (COVID-19): a pictorial review. *Eur Radiol* **30**, 4381-4389, doi:10.1007/s00330-020-06801-0 (2020).

19 King, T. E., Jr. *et al.* A phase 3 trial of pirfenidone in patients with idiopathic pulmonary fibrosis. *N. Engl. J. Med* **370**, 2083-2092 (2014).

20 Wollin, L., Maillet, I., Quesniaux, V., Holweg, A. & Ryffel, B. Antifibrotic and anti-inflammatory activity of the tyrosine kinase inhibitor nintedanib in experimental models of lung fibrosis. *J. Pharmacol. Exp. Ther* **349**, 209-220 (2014).

21 Raghu, G. & Selman, M. Nintedanib and pirfenidone. New antifibrotic treatments indicated for idiopathic pulmonary fibrosis offer hopes and raises questions. *Am. J. Respir. Crit Care Med* **191**, 252-254 (2015).

22 Somogyi, V. *et al.* The therapy of idiopathic pulmonary fibrosis: what is next? *Eur Respir Rev* **28**, doi:10.1183/16000617.0021-2019 (2019).

23 Sjoberg, A. P., Trouw, L. A. & Blom, A. M. Complement activation and inhibition: a delicate balance. *Trends Immunol* **30**, 83-90 (2009).

24 Ricklin, D., Hajishengallis, G., Yang, K. & Lambris, J. D. Complement: a key system for immune surveillance and homeostasis. *Nat. Immunol* **11**, 785-797 (2010).

25 Carroll, M. V. & Sim, R. B. Complement in health and disease. *Adv. Drug Deliv. Rev* **63**, 965-975 (2011).

26 Wong, E. K. S. & Kavanagh, D. Diseases of complement dysregulation-an overview. *Semin Immunopathol* **40**, 49-64, doi:10.1007/s00281-017-0663-8 (2018).

27 Brown, K. M., Sacks, S. H. & Sheerin, N. S. Mechanisms of disease: the complement system in renal injury--new ways of looking at an old foe. *Nat. Clin. Pract. Nephrol* **3**, 277-286 (2007).

28 Pandya, P. H. & Wilkes, D. S. Complement system in lung disease. *Am J Respir Cell Mol Biol* **51**, 467-473, doi:10.1165/rcmb.2013-0485TR (2014).

29 Kou, W. *et al.* High complement protein C1q levels in pulmonary fibrosis and non-small cell lung cancer associated with poor prognosis. *BMC Cancer* **22**, 110, doi:10.1186/s12885-021-08912-3 (2022).

30 Ogawa, T., Shichino, S., Ueha, S., Ogawa, S. & Matsushima, K. Complement protein C1q activates lung fibroblasts and exacerbates silica-induced pulmonary fibrosis in mice. *Biochem Biophys Res Commun* **603**, 88-93, doi:10.1016/j.bbrc.2022.02.090 (2022).

31 Nayak, A., Pednekar, L., Reid, K. B. & Kishore, U. Complement and non-complement activating functions of C1q: a prototypical innate immune molecule. *Innate Immun* **18**, 350-363, doi:10.1177/1753425910396252 (2012).

32 Naito, A. T. *et al.* Complement C1q activates canonical Wnt signaling and promotes aging-related phenotypes. *Cell* **149**, 1298-1313 (2012).

33 Sumida, T. *et al.* Complement C1q-induced activation of β-catenin signalling causes hypertensive arterial remodelling. *Nat Commun* **6**, 6241, doi:10.1038/ncomms7241 (2015).

34 Son, M. *et al.* C1q and HMGB1 reciprocally regulate human macrophage polarization. *Blood* **128**, 2218-2228 (2016).

35 Spivia, W., Magno, P. S., Le, P. & Fraser, D. A. Complement protein C1q promotes macrophage anti-inflammatory M2-like polarization during the clearance of atherogenic lipoproteins. *Inflamm Res* **63**, 885-893, doi:10.1007/s00011-014-0762-0 (2014).

36 Vogel, D. Y. *et al.* Macrophages migrate in an activation-dependent manner to chemokines involved in neuroinflammation. *J Neuroinflammation* **11**, 23, doi:10.1186/1742-2094-11-23 (2014).

37 Park, M. D., Silvin, A., Ginhoux, F. & Merad, M. Macrophages in health and disease. *Cell* **185**, 4259-4279, doi:10.1016/j.cell.2022.10.007 (2022).

38 Wynn, T. A. & Vannella, K. M. Macrophages in Tissue Repair, Regeneration, and Fibrosis. *Immunity* **44**, 450-462 (2016).

39 Zhang, L. *et al.* Macrophages: friend or foe in idiopathic pulmonary fibrosis? *Respir. Res* **19**, 170-0864 (2018).

40 Cheng, P., Li, S. & Chen, H. Macrophages in Lung Injury, Repair, and Fibrosis. *Cells* **10**, doi:10.3390/cells10020436 (2021).

41 Kamiya, M. *et al.* Immune mechanisms in fibrotic interstitial lung disease. *Cell* **187**, 3506-3530, doi:10.1016/j.cell.2024.05.015 (2024).

42 Zhang, F. *et al.* Reprogramming of profibrotic macrophages for treatment of bleomycin-induced pulmonary fibrosis. *EMBO Mol. Med* **12**, e12034 (2020).

43 Xu, Y. *et al.* Single-cell RNA sequencing identifies diverse roles of epithelial cells in idiopathic pulmonary fibrosis. *JCI. Insight* **1**, e90558 (2016).

44 Hong, Q. *et al.* Complement C1q activates tumor suppressor WWOX to induce apoptosis in prostate cancer cells. *PLoS One* **4**, e5755, doi:10.1371/journal.pone.0005755 (2009).

45 Kaur, A. *et al.* Human C1q Induces Apoptosis in an Ovarian Cancer Cell Line via Tumor Necrosis Factor Pathway. *Front Immunol* **7**, 599, doi:10.3389/fimmu.2016.00599 (2016).

46 Roque, W. & Romero, F. Cellular metabolomics of pulmonary fibrosis, from amino acids to lipids. *Am J Physiol Cell Physiol* **320**, C689-c695, doi:10.1152/ajpcell.00586.2020 (2021).

47 Parimon, T., Yao, C., Stripp, B. R., Noble, P. W. & Chen, P. Alveolar Epithelial Type II Cells as Drivers of Lung Fibrosis in Idiopathic Pulmonary Fibrosis. *Int J Mol Sci* **21**, doi:10.3390/ijms21072269 (2020).

48 Wang, Y., Wang, L., Ma, S., Cheng, L. & Yu, G. Repair and regeneration of the alveolar epithelium in lung injury. *Faseb j* **38**, e23612, doi:10.1096/fj.202400088R (2024).

49 Rodriguez, L. R. *et al.* Impaired AMPK Control of Alveolar Epithelial Cell Metabolism Promotes Pulmonary Fibrosis. *bioRxiv*, doi:10.1101/2024.03.26.586649 (2024).

50 Zhu, W., Tan, C. & Zhang, J. Alveolar Epithelial Type 2 Cell Dysfunction in Idiopathic Pulmonary Fibrosis. *Lung* **200**, 539-547, doi:10.1007/s00408-022-00571-w (2022).

51 Gschwend, J. *et al.* Alveolar macrophages rely on GM-CSF from alveolar epithelial type 2 cells before and after birth. *J Exp Med* **218**, doi:10.1084/jem.20210745 (2021).

52 Westphalen, K. *et al.* Sessile alveolar macrophages communicate with alveolar epithelium to modulate immunity. *Nature* **506**, 503-506 (2014).

53 Planté-Bordeneuve, T., Pilette, C. & Froidure, A. The Epithelial-Immune Crosstalk in Pulmonary Fibrosis. *Front Immunol* **12**, 631235, doi:10.3389/fimmu.2021.631235 (2021).

54 Liu, X., Boyer, M. A., Holmgren, A. M. & Shin, S. Legionella-Infected Macrophages Engage the Alveolar Epithelium to Metabolically Reprogram Myeloid Cells and Promote Antibacterial Inflammation. *Cell Host Microbe* **28**, 683-698.e686, doi:10.1016/j.chom.2020.07.019 (2020).
